# Dynamics of biomass and carbon sequestration across a chronosequence of *Caragana intermedia* plantations on alpine sandy land

**DOI:** 10.1038/s41598-018-30595-3

**Published:** 2018-08-20

**Authors:** Qingxue Li, Zhiqing Jia, Lili Feng, Lingxianzi He, Kaiyue Yang

**Affiliations:** 1 Institute of Desertification Studies, Chinese Academy of Forestry, Beijing, 100091 China; 2 Qinghai Gonghe Desert Ecosystem Research Station, Qinghai, 813005 China; 3 School of Mining and Geomatics Engineering, Hebei University of Engineering, Hebei, Handan 056038 China

## Abstract

Considering the variations in carbon concentrations among different plant components can significantly improve carbon storage estimates. The aim of this study was to estimate the biomass and carbon storage and sequestration in *Caragana intermedia* Kuang et H. C. Fu plantations for six different ages. The biomass and carbon sequestration in shrub biomass were quantified using a destructive method that involved analysing the carbon concentrations in the leaves, branches, stem bark, stem wood, roots > 5 mm, 2 mm < roots ≤ 5 mm, and roots ≤ 2 mm. The biomass and biomass carbon density of the *C. intermedia* plantations increased with the age of the stands. The biomass carbon density of the woody components was at its maximum in the 30-year-old plantation (14.27 ± 2.71 t·ha^−1^), indicating that *C. intermedia* plantations in alpine sandy land are an important carbon pool. The carbon accumulation rate of the woody components was higher during the early stages of plantation development. The carbon concentrations differed significantly among the components but changed only slightly with the stand age. The weighted mean carbon concentration of the woody components, which was found to be 44%, must be considered when estimating the long-term carbon pools in *C. intermedia* plantations.

## Introduction

Forests account for approximately one-third of the Earth’s land area, storing 80% of the total aboveground carbon in terrestrial ecosystems and 40% of the belowground carbon^1,2^, and therefore, they perform important roles in maintaining the global carbon balance^3^ and mitigating global climate change^4^. The precise estimation of forest carbon stocks and the prediction of the carbon potential on regional and global scales are important research topics^5^. Many researchers have assumed a carbon fraction of 50% for converting forest vegetation biomass into carbon^6,7^. However, Sean and Martin found that a constant value of 50% for the carbon concentration in tree biomass is not accurate^8^. Therefore, it is necessary to use the carbon concentrations of specific components to obtain better carbon estimates for forest ecosystem wood and achieve better precision^9,10^.

Many studies that have estimated carbon stocks have focused on tree species^6,9–11^, but little attention has been paid to carbon stock estimates for shrub species^12^. Shrubs exhibit great species richness, vitality, sprouting ability, productivity and adaptability and play extremely important roles not only in the community succession process but also in regional environmental protection^13^. Carbon uptake by shrubs accounts for approximately one-third of the total carbon sink^14^, and shrubs represent a large biomass and carbon pool that is usually underestimated in carbon storage assessments^15^.

Arid and semi-arid lands cover nearly 45% of the global terrestrial area^16,17^, and the biomass per unit area is normally low in these regions. However, their vast area provides them with a significant role as a carbon pool^18,19^. In these regions, desertification is one of the primary types of land degradation and results from various factors^20^. The world’s average desert biomass carbon density is only 3.5 t·ha^−1^, which is much smaller than that of forests and grasslands^21^. Tao and Zhang^22^ reported that the vegetation types of temperate semi-shrubs and dwarf semi-shrubs exhibited the highest carbon storage capacities, and the average biomass carbon density of arid regions in Central Asia is 1.837 t·ha^−1^. China has been severely affected by desertification, and the desertified land area accounts for 45% of the total territory^23^. Revegetation is an effective way to combat and control desertification^24^, and shrub species have been widely favoured in vegetation restoration efforts due to their tolerance of harsh environments^25,26^. In the context of global climate warming, studies on the carbon fixation function of vegetation in desert areas are very important^27^, especially in terms of carbon sequestration by shrubs after vegetation restoration^23^.

Most leguminous shrubs in the genus *Caragana* have strong drought resistance capabilities and fast growth, and they are important xerophytic shrub species for windbreaks, sand fixation, and soil and water conservation. For example, *Caragana* (Lam.) was intentionally introduced to the northern Great Plains of North America for erosion control^28^. *Caragana microphylla* Lam. is a pioneer leguminous shrub species for vegetation re-establishment that is widely distributed throughout the semi-fixed and fixed sandy lands of the Horqin region^29^. *Caragana jubata* (Pall.) thrives well under the extreme cold of the high altitudes of the Himalayas, and hence, this plant is expected to be a source of genes that might play an important role in tolerating low temperatures^30^. *Caragana intermedia* Kuang et H. C. Fu is a native desert shrub that is widespread in the desert land of western and north-western China^31^. The typical distribution range of *C. intermedia* is in the desert steppe area of the Mongolia Plateau, the typical steppe area and desert steppe area of the Erdos Plateau, and the typical steppe area and steppe area of the North Loess Plateau, forming a common *C. intermedia* sandy land shrub community^32^.

The Gonghe Basin of the Tibetan Plateau is one of the regions that has been severely affected by desertification, and the desertified area accounts for 91% of the area of the whole basin^33^. The establishment of sand barrier enclosures and the planting of shrubs on shifting sand dunes are effective methods for combating and controlling desertification in this region^14,34^. Large shrub plantations have been established with the help of straw checkerboards since 1958^33^. *C. intermedia* was widely planted on shifting sand dunes after the erection of the straw checkerboards due to its tolerance to drought, cold, and poor habitats and for its rapid growth. For example, some studies of *C. intermedia* plantations in this region have showed that *C. intermedia* development influences the chemical and physical properties of soil^26,35,36^; it can increase water use efficiency to resist drought stress^37^, and the primary water sources are affected by its root distribution^38^. In addition, the root system distributions of the *C. intermedia* plantations showed that the absorptive roots (diameter ≤ 1 mm) of 5-, 9- and 25-year-old plantations are primarily distributed in the 10–30 cm, 10–50 cm and 10–60 cm soil layers, respectively, whereas the conductive roots (diameter >1 mm) primarily appear in the 10–50 cm, 10–60 cm and 10–90 cm soil layers, respectively^39^. However, there is little information on the biomass accumulation and carbon sequestration capacity of *C. intermedia* plantations, and the effects of these functions on the carbon sequestration capacity of shrubland should not be neglected. Therefore, a better understanding of the biomass carbon density and the carbon sequestration capacity of *C. intermedia* plantations could be helpful for improving the precision of shrubland carbon sequestration evaluations, and this information is also important for the sustainable management of sand-fixing plantations.

Therefore, we performed a sampling campaign of different shrub ages on sand dunes in *C. intermedia* plantations to test three general hypotheses as follows: (1) the carbon sequestration of *C. intermedia* plantations would increase as the stand aged, and the carbon accumulation rate would decrease as the stand aged; (2) there is no obvious change in carbon concentrations in the shrub components with the stand age; and (3) using the carbon fraction of 50% for converting *C. intermedia* biomass into carbon will overestimate the carbon stocks.

## Results

### Biomass and its distribution into components

The component, aboveground, belowground and total biomass for the *C. intermedia* plantation stands significantly differ with the plant age (*P* < 0.05) (Table 1). The branch, stem wood, roots > 5 mm, 2 mm < roots ≤ 5 mm, belowground and total biomass showed the same trend with an increase in the stand age following the natural order of increases of 3-year-old stand < 5-year-old stand < 8-, 10-, and 16-year-old stands < 30-year-old stand, but there was no consistent difference among the 8-, 10- and 16-year-old stands. The aboveground biomass for the 16-year-old stand was only slightly higher than that recorded for the 3-year-old stand and lower than that for stands of other ages; however, the belowground biomass for the 16-year-old stand was slightly lower than that recorded for the 30-year-old stand and higher than that for stands of other ages. The woody component biomass (leafless) of the *C. intermedia* plantations increased as the stand aged, peaking in the 30-year-old plantation at 32.26 ± 6.15 t·ha^−1^.

**Table 1:** Biomass of *C. intermedia* plantation stands of different ages (t·ha^−1^).

	Stand age (Years)					
	3	5	8	10	16	30
Leaf	1.49 ± 0.26a	2.51 ± 0.20bc	2.87 ± 0.14c	2.21 ± 0.21b	1.23 ± 0.05a	2.41 ± 0.19bc
Branch	2.00 ± 0.36a	2.95 ± 0.23b	6.49 ± 0.03d	5.10 ± 0.61 cd	3.75 ± 0.11bc	11.53 ± 2.64e
Stem bark	0.13 ± 0.02a	0.23 ± 0.06a	0.39 ± 0.01b	0.42 ± 0.05b	0.22 ± 0.01a	0.79 ± 0.13c
Stem wood	0.34 ± 0.07a	0.46 ± 0.11a	1.00 ± 0.04b	0.99 ± 0.13b	0.54 ± 0.01a	1.66 ± 0.30c
Roots > 5 mm	4.16 ± 0.70a	6.25 ± 0.49b	11.02 ± 0.10c	7.33 ± 0.49b	12.59 ± 0.30c	14.82 ± 2.47c
2 mm < Roots ≤ 5 mm	0.96 ± 0.12a	1.25 ± 0.08ab	1.45 ± 0.06ab	1.61 ± 0.27b	1.84 ± 0.14bc	2.53 ± 0.53c
Roots ≤ 2 mm	0.35 ± 0.02a	0.81 ± 0.06c	0.60 ± 0.04b	0.42 ± 0.05a	0.85 ± 0.04c	0.93 ± 0.15c
Aboveground biomass	3.96 ± 0.70a	6.16 ± 0.34bc	10.75 ± 0.10d	8.73 ± 0.82 cd	5.74 ± 0.18b	16.39 ± 3.23e
Belowground biomass	5.47 ± 0.82a	8.32 ± 0.61b	13.08 ± 0.17c	9.35 ± 0.78b	15.29 ± 0.43c	18.28 ± 3.13c
Woody components biomass	7.94 ± 1.26a	11.96 ± 0.82ab	20.95 ± 0.23c	15.87 ± 1.17abc	19.80 ± 0.55bc	32.26 ± 6.15d
Total biomass	9.43 ± 1.52a	14.47 ± 0.96b	23.83 ± 0.15c	18.08 ± 1.33bc	21.03 ± 0.60c	34.67 ± 6.34d

Values followed by different lowercase letters indicate significant differences among stand ages according to Duncan’s multiple range test (*P* < 0.05). The values are the means ± SE (n = 3).

**Figure 1:**
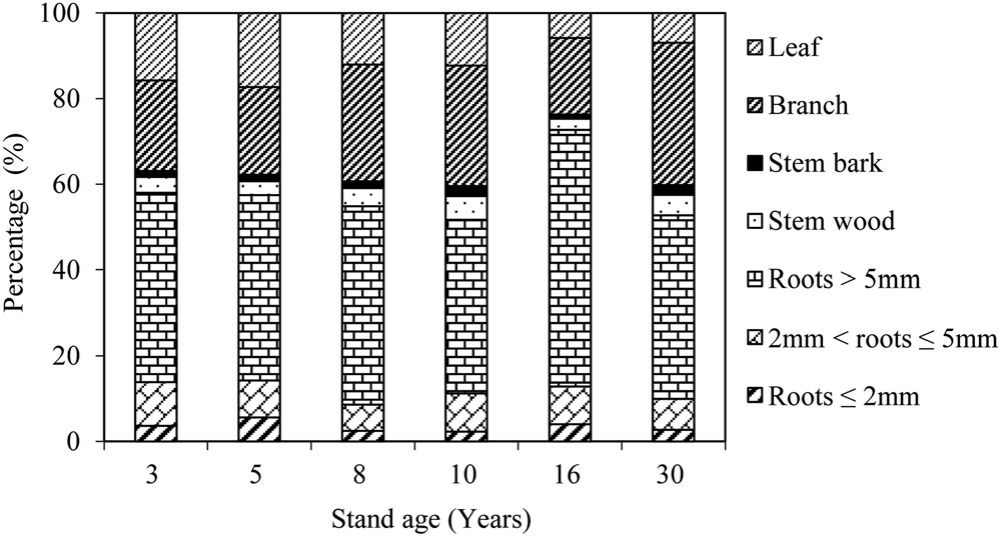
Proportion of the biomass of different components in the *C. intermedia* plantation stands at different ages (%).

The proportion of leaf biomass decreased and the proportion of the biomass of woody components increased as the stand age increased (Fig. 1). The proportions of branch, stem bark and stem wood biomass showed the same increasing trend with the stand age in the 3-, 5-, 8-, and 10-year-old stands, declining in the 16-year-old stand, and increasing again in the 30-year-old stand. The root biomass proportion showed the opposite trend, declining with the stand age in the 3-, 5-, 8-, and 10-year-old stands, increasing in the 16-year-old stand, and declining again in the 30-year-old stand. The percentage of biomass in woody components that accounted for the total shrub biomass of all the stand age plantations ranged from 83–94%, and the average percentage was 88 ± 2%. The percentage of belowground biomass that accounted for the biomass of woody components of all ages of stand plantations ranged from 57–77%, and the average percentage was 66 ± 3%.

### Carbon concentrations

The carbon concentrations of the *C. intermedia* components in the stands of the same age were significantly different (*P* < 0.05) (Table 2). The carbon concentrations in the components did not change significantly with the stand age. The carbon concentrations of the *C. intermedia* components ranged from 41% to 46%, and the weighted average carbon concentration of the wood components for the six stand ages was 44%; this result could be considered the carbon concentration of the whole shrubs for long-term carbon pool estimations in *C. intermedia* plantations.

**Table 2:** Biomass carbon concentrations for *C. intermedia* plantation stands of different ages (%).

	Stand age (Years)						Average
	3	5	8	10	16	30	
Leaf	45.62 ± 0.07c	45.39 ± 0.48c	45.35 ± 0.40c	45.49 ± 0.29d	44.59 ± 0.37 cd	45.27 ± 0.40e	45.29 ± 0.33
Branch	42.66 ± 0.23b	42.64 ± 0.65b	43.28 ± 0.27b	42.23 ± 0.21b	42.91 ± 0.17b	43.91 ± 0.23bc	42.94 ± 0.29
Stem bark	45.39 ± 0.36c	45.45 ± 0.22c	45.60 ± 0.06c	45.45 ± 0.63d	45.45 ± 0.23d	46.07 ± 0.14 f	45.57 ± 0.27
Stem wood	41.91 ± 0.34a	41.39 ± 0.28a	41.41 ± 0.45a	41.53 ± 0.56ab	41.68 ± 0.31a	42.33 ± 0.55a	41.71 ± 0.42
Roots > 5 mm	45.62 ± 0.07c	45.45 ± 0.22c	45.49 ± 0.29c	45.57 ± 0.41d	45.41 ± 1.05d	44.65 ± 0.48d	45.37 ± 0.42
2 mm < Roots ≤ 5 mm	42.66 ± 0.23b	41.39 ± 0.28a	41.08 ± 0.94a	44.59 ± 0.37c	43.91 ± 0.23c	44.27 ± 0.03 cd	42.98 ± 0.35
Roots ≤ 2 mm	45.39 ± 0.36c	42.64 ± 0.65b	45.60 ± 0.06c	41.19 ± 0.51a	44.94 ± 0.23d	43.34 ± 0.28b	43.85 ± 0.35
Weighted average (leafless)	44.34 ± 0.05	43.99 ± 0.15	44.31 ± 0.18	44.02 ± 0.13	44.66 ± 0.39	44.23 ± 0.18	44.26 ± 0.09
Weighted average (whole shrub)	44.54 ± 0.04	44.23 ± 0.13	44.44 ± 0.19	44.20 ± 0.12	44.66 ± 0.36	44.31 ± 0.16	44.40 ± 0.07

Values followed by different lowercase letters indicate a significant difference among the components according to Duncan’s multiple range test (*P* < 0.05). The values are the means ± SE (n = 3).

### Biomass carbon density and its distribution in different components

The component, aboveground, belowground and total biomass carbon densities for the *C. intermedia* plantation stands of different ages were significantly different (*P* < 0.05) (Table 3). The branch, stem wood, roots > 5 mm, 2 mm < roots ≤ 5 mm, belowground and total biomass carbon densities showed the same trend as the stand age increased, with 3-year-old stand < 5-year-old stand < 8-, 10-, and 16-year-old stands < 30-year-old stand, but there was no consistent difference among the 8-, 10- and 16-year-old stands. The aboveground biomass carbon density for the 16-year-old stand was only slightly higher than that found for the 3-year stand and lower than that found for the stands of other ages. The belowground biomass carbon density for the 16-year-old stand was slightly lower than that recorded for the 30-year-old stand and higher than that for the stands of other ages. The biomass carbon density for the woody components of *C. intermedia* plantations increased as the stand aged, peaking in the 30-year-old plantation at 14.27 ± 2.71 t·ha^−1^.

**Table 3:** Biomass carbon density for *C. intermedia* plantation stands of different ages (t·ha^−1^).

	Stand age (Years)					
	3	5	8	10	16	30
Leaf	0.68 ± 0.21a	1.14 ± 0.15bc	1.30 ± 0.12c	1.01 ± 0.16b	0.55 ± 0.04a	1.09 ± 0.16bc
Branch	0.85 ± 0.28a	1.26 ± 0.17ab	2.81 ± 0.03 cd	2.15 ± 0.44d	1.61 ± 0.09bc	5.07 ± 2.02e
Stem bark	0.06 ± 0.02a	0.11 ± 0.05a	0.18 ± 0.01b	0.19 ± 0.05b	0.10 ± 0.01a	0.37 ± 0.10c
Stem wood	0.14 ± 0.05a	0.19 ± 0.08a	0.42 ± 0.03b	0.42 ± 0.10b	0.22 ± 0.01a	0.70 ± 0.23c
Roots > 5 mm	1.90 ± 0.55a	2.84 ± 0.38b	5.02 ± 0.07c	3.34 ± 0.36b	5.72 ± 0.34c	6.61 ± 1.89c
2 mm < Roots ≤ 5 mm	0.41 ± 0.09a	0.52 ± 0.06ab	0.60 ± 0.04ab	0.71 ± 0.20b	0.81 ± 0.11bc	1.12 ± 0.41c
Roots ≤ 2 mm	0.16 ± 0.01a	0.35 ± 0.04bc	0.27 ± 0.03b	0.17 ± 0.04a	0.38 ± 0.03c	0.40 ± 0.11c
Aboveground biomass	1.73 ± 0.31a	2.69 ± 0.14bc	4.70 ± 0.06d	3.76 ± 0.35 cd	2.48 ± 0.08b	7.22 ± 1.44e
Belowground biomass	2.47 ± 0.37a	3.70 ± 0.27b	5.88 ± 0.06c	4.22 ± 0.33b	6.91 ± 0.26c	8.14 ± 1.38c
Woody components biomass	3.52 ± 0.56a	5.26 ± 0.34ab	9.28 ± 0.09c	6.98 ± 0.50abc	8.85 ± 0.32bc	14.27 ± 2.71d
Total biomass	4.20 ± 1.18a	6.40 ± 0.70b	10.59 ± 0.14c	7.99 ± 0.99bc	9.40 ± 0.59c	15.36 ± 4.86d

Values followed by different lowercase letters indicate significant differences among stand ages according to Duncan’s multiple range test (*P* < 0.05). The values are the means ± SE (n = 3).

**Figure 2:**
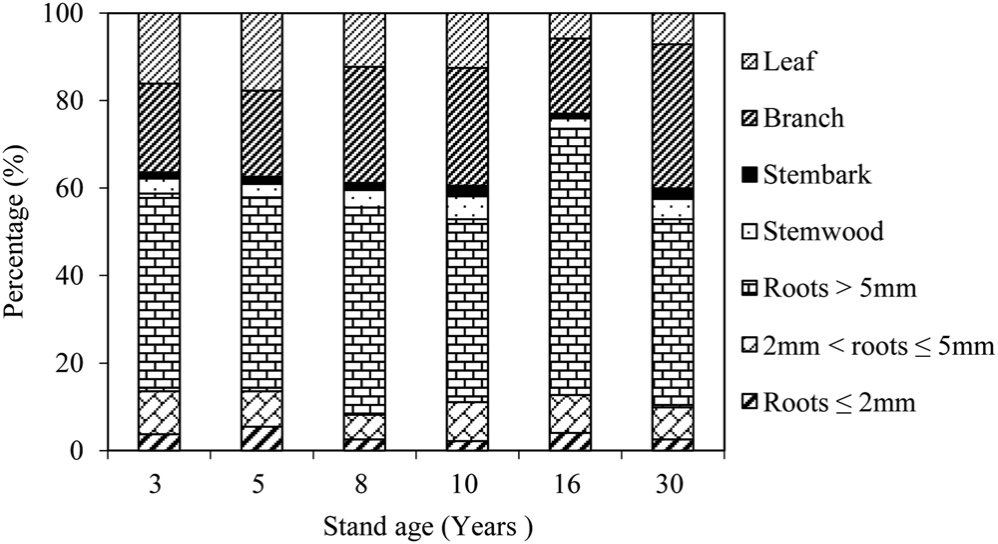
Proportion of the biomass carbon density of different components in the *C. intermedia* plantation stands of different ages (%).

The proportion of biomass carbon density in the leaves decreased and it increased in the woody components as the stand age increased (Fig. 2). The proportions of branch, stem bark and stem wood biomass carbon density showed the same trend as the stand age increased, increasing in the 3-, 5-, 8-, and 10-year-old stands, declining in the 16-year-old stand, and increasing again in the 30-year-old stand. The proportion of the root biomass carbon density showed the opposite pattern, declining in the 3-, 5-, 8-, and 10-year-old stands, increasing in the 16-year-old stand, and declining again in the 30-year-old stand. The percentage of biomass carbon density in woody components that accounted for the total shrub biomass carbon density of all ages of stand plantations ranged from 82–94%, and the average percentage was 88 ± 2%. The percentage of belowground biomass carbon density that accounted for the biomass carbon density of woody components of all the stand age plantations ranged from 57–78%, and the average percentage was 67 ± 3%.

### Carbon accumulation rate of woody components

The carbon accumulation rates of the woody components estimated in the 3-, 5- and 8-year-old stands (1.17 ± 0.19 t·ha^−1^·yr^−1^, 1.05 ± 0.07 t·ha^−1^·yr^−1^ and 1.16 ± 0.01 t·ha^−1^·yr^−1^, respectively) were significantly higher than those in the 10-, 16- and 30-year-old stands (0.70 ± 0.05 t·ha^−1^·yr^−1^, 0.55 ± 0.02 t·ha^−1^·yr^−1^ and 0.48 ± 0.09 t·ha^−1^·yr^−1^, respectively).

## Discussion

The biomass quantification of woody plants has been recognized as a crucial step in the calculation of forest biomass and carbon stocks^40^. In our results, the biomass and carbon density of the measured *C. intermedia* shrubs increased as the stand age increased, which indicates that shrubs continuously absorb CO_2_ during the photosynthesis and subsequently store the resulting carbohydrates in their biomass. Therefore, the stand age has positive impacts on the forest ecosystem and nutrient cycling^10,41^. However, each species has its own lifespan, and the biomass and carbon sequestration capacity peak in the mature stage and decrease afterwards^42^. In this study, the increase in the total biomass and biomass carbon density of *C. intermedia* plantations with the stand age indicated that the 30-year-old plantation was in the mature or premature stage, which is characterized by a relatively significant carbon sequestration capacity. The biomass carbon density of the woody components of 30-year-old *C. intermedia* plantations reached 14.27 ± 2.71 t·ha^−1^, which was much higher than the global average desert biomass carbon density of 3.5 t·ha^−1 ^^21^. This result shows that the *C. intermedia* plantation in alpine sandy land is an important carbon pool, and if all of the sandy land is restored, it will have great potential for carbon sequestration. Deng *et al*. reported that the carbon pools in the biomass of *Caragana korshinskii* plantations on the Loess Plateau increased with the stand age and reached 14.11 ± 1.54 Mg·ha^−1^ at 35 years and then declined^42^. Wang *et al*. reported that the plant biomass carbon of *Caragana stenophylla* was 5.88 Mg·ha^−1^ in arid regions of Xinjiang^43^. In the arid zone of India, the carbon density of *Calligonum polygonoides* L. ranged between 0.37 and 1.84 t·ha^−1 ^^44^, which was obviously lower than it was in our study. The 16-year-old stand showed a greater root biomass proportion (77%), which might be attributed to the accelerated development of root systems reaching its maximum by this age, and further studies are required to confirm the findings.

Osone and Tateno reported that plants allocate more biomass to underground organs to absorb more soil moisture or nutrients when water or nutrients are the primary limiting factors^45^. In our study, the average percentages of the root biomass and the biomass carbon density of the total values for the woody components were 66 ± 3% and 67 ± 3%, respectively, demonstrating that the *C. intermedia* roots were the dominant organs. This result probably occurred because *C. intermedia* needs to produce more roots to absorb water and nutrients to survive in barren alpine sandy land. Deng *et al*. reported that the ratio of roots to the total biomass of *C. korshinskii* plantations on the Loess Plateau was higher during the mature and post-mature stages, at 58.7%^42^. Wang *et al*. found that the underground plant biomass carbon densities of the *Caragana stenophylla*, *Ephedra przewalskii*, *Haloxylon persicum*, *Calligonum mongolicum*, *Nitraria tangutorum*, *Anabasis elatior* and *Anabasis brevifolia* shrub communities were higher than those of the above ground communities in Xinjiang^43^. Qiu *et al*. also found that the root biomass of *Spiraea hypericifolia* L. accounts for 72.9% of the total plant biomass in the forests of the Tianshan Mountains in China^46^.

The carbon accumulation rate of woody components was higher during the early stages of plantation development, and the 3-year-old stand reached 1.17 t·ha^−1^·yr^−1^, which is consistent with the results of Dixon *et al*., who reported that young forests take up carbon dioxide at relatively high rates^1^. The study about the biomass carbon sequestration of a *C. korshinskii* plantation on the Loess Plateau also found that the annual rate of biomass carbon accumulation decreased with the plantation development and that of young plantations reached 0.76 Mg·ha^−1^·yr^−1 ^^42^. Francis Justine *et al*. also found that changes in the biomass accumulation rate of *Pinus massoniana* decreased significantly with the forest age and that the highest accumulation rate occurred during the early stages of forest growth^10^.

The weighted average carbon concentration of the wood components was 44%, and this result is supported by those of Fonseca *et al*., who reported carbon fractions in the range of 0.40 to 0.45^11^. These results clearly show that the assumed carbon concentration of 50%^6,7^ for the components was generally higher than the measured carbon concentrations in the *C*. *intermedia* plantations and may result in an approximate 13% overestimation of the carbon sequestration of *C. intermedia* plantations. The high variation in the carbon concentrations among components from a single species may be affected by a range of factors, such as the sample chemical composition and origin and the selected analytical methods^47^. However, the carbon concentrations did not differ significantly with the stand age, which is consistent with the assumption that the ability of wood to store carbon in mature stands is the same as that in young stands^10^. Our findings are consistent with those of previous studies conducted by Maiti *et al*., who found that the carbon concentration differed significantly among tree organs, while there was no significant difference among the ages^48^.

In summary, the biomass and biomass carbon density of *C. intermedia* plantations increased as the stand age increased, and the 30-year-old plantation showed the highest biomass carbon stock, although the carbon accumulation rate peaked in younger, 3- to 8- year-old stands. The biomass carbon density of woody components from *C. intermedia* plantations reached 14.27 ± 2.71 t·ha^−1^, which shows that the *C. intermedia* plantations in alpine sandy land are an important carbon pool. The carbon accumulation rate of wood components was higher during the early stages of plantation development, and the 3-year-old stand reached 1.17 t·ha^−1^·yr^−1^. The roots presented the primary biomass and biomass carbon stocks. The carbon concentrations of the *C. intermedia* components in stands of the same age were significantly different, whereas the carbon concentrations in the components hardly changed with the stand age. Therefore, measuring the carbon concentrations once in the components of *C. intermedia* would suffice for its entire lifetime when estimating the plantation carbon sequestration. Using a constant value of 50% for the carbon concentration in *C. intermedia* biomass may result in an approximate 13% overestimation of the carbon sequestration of *C. intermedia* plantations. Therefore, the weighted mean carbon concentration of the wood components of 44% rather than the generic value of 50% must be considered for long-term carbon pool estimations in *C. intermedia* plantations.

## Materials and Methods

### Study site

The study was carried out at the desertification-combating experimental site of the Qinghai Gonghe Desert Ecosystem Research Station (99°45′–100°30′E, 36°03′–36°40′N and altitude 2871 m), and the total area of the experimental site is 1094 ha. The station is located in the Gonghe Basin of the north-eastern Tibetan Plateau, and it is a part of the Chinese Desert Ecosystem Research Network (CDERN) of the State Forestry Administration of P.R. China. The station was constructed by the Chinese Academy of Forestry and the Desertification Combating Station of Qinghai Province. The mean annual temperature is 2.4 °C, the mean annual precipitation is 246.3 mm, and the mean annual potential evaporation is 1716.7 mm. The mean annual number of windy days is 50.6 days, and the mean annual number of sandstorm days is 20.7 days. The mean annual wind speed is 2.7 m·s^−1^, and the maximum wind speed reaches 40 m·s^−1^. Chestnut soil and brown soil are the zonal soils in the study area, while aeolian, meadow and bog soils are the azonal soils. The primary vegetation type is sand-fixing plantation, which includes the tree species *Populus simonii* Carr. and *Populus cathayana* Rehd. and the shrub species *C. intermedia*, *Caragana korshinskii* Kom., *Salix cheilophila* Schneid., *Hippophae rhamnoides* L., and *Salix psammophila* C. Wang et Chang Y. Yang^27,31^. Zhang *et al*. reported that *C. intermedia* is a variant of *C. korshinskii*^44^. In the study area, the morphological characteristics of these two species are noticeably different, and *C. intermedia* plantations are distributed more widely; therefore, they were selected for this study.

### Experimental design and sampling

**Figure 3:**
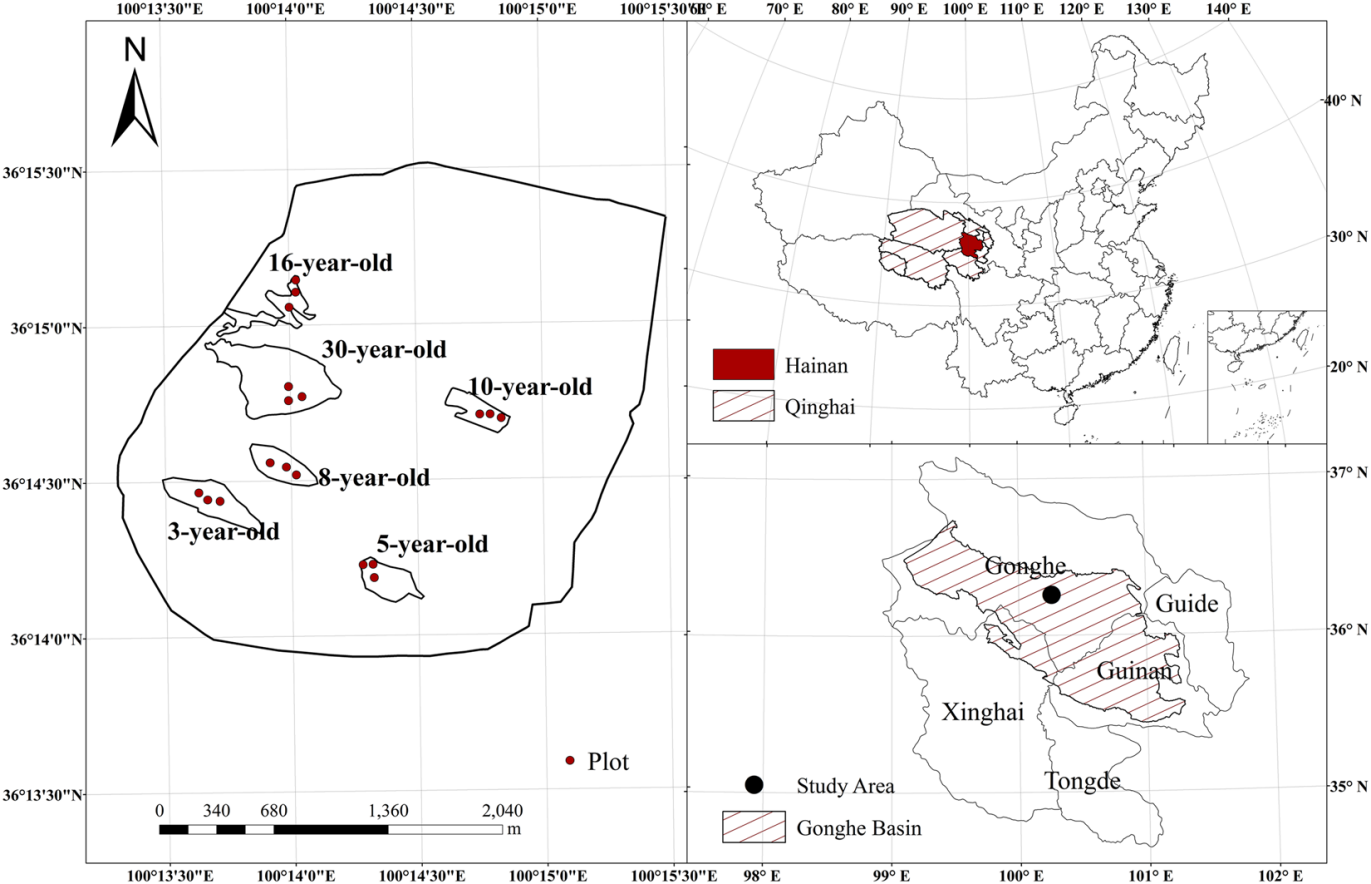
The sampling location of *C. intermedia* plantations in the Gonghe Basin, Qinghai Province, China.

Our study was conducted in mid-August 2016, when the biomass of the *C. intermedia* stands peaked. All the *C. intermedia* plantations in the study area were sown. Therefore, each shrub grows from multiple seeds. In addition, *C. intermedia* has strongly asexual germination, resulting in multiple stems, and all the stems from different seeds grow in a staggered fashion. Three representative plots (10 × 10 m) with slopes within 15° were established in 3-, 5-, 8-, 10-, 16- and 30-year-old mono-species plantations of *C. intermedia* as the sampling sites (Fig. 3). The plots were more than 50 m apart. The areas of the 3-, 5-, 8-, 10-, 16- and 30-year-old stands are 8.71, 5.72, 5.65, 4.82, 5.67 and 20.91 ha, respectively. In all the plantations, straw checkerboards (1 × 1 m) were established on shifting sand dunes for sand fixation and then sowed with *C. intermedia*. In each plot, the morphological characteristics of 10 plants were measured, including the maximum height (H), basal diameter, crown diameter (maximum diameter of the shrub canopy in the northeast-southwest (C_NE-SW_) and northwest-southeast (C_NW-SE_) directions), and ground diameter (the maximum diameter of the area shrub shoots emerged at ground level in the northeast-southwest (G_NE-SW_) and northwest-southeast (G_NW-SE_) directions). *Caragana intermedia* is a shrub with multiple stems; thus, the stems were categorized into three diameter classes (maximum, medium and minimum), and a representative stem basal diameter was measured first to calculate a mean value of each shrub’s basal diameter (D). One standard shrub was selected from each plot according to the mean morphological characteristics for different *C. intermedia* plantation ages, for a total of 18 plants harvested for biomass measurements. The morphological characteristics of the standard shrubs are shown in Table 4.

**Table 4:** The morphological characteristics of *C. intermedia* plantation stands of different ages.

Stand age (Years)	Height (m)	Basal diameter (cm)	Crown diameter (m)		Ground diameter (m)		Array spacing (m)
			**C**_NE-SW_	C_NW-SE_	G_NE-SW_	G_NW-SE_	
3	0.80 ± 0.01	0.53 ± 0.01	1.13 ± 0.03	1.00 ± 0.00	0.42 ± 0.02	1.00 ± 0.00	Sown in lines with line spacing of 1 m
5	1.12 ± 0.04	0.69 ± 0.01	1.23 ± 0.03	1.00 ± 0.00	0.50 ± 0.03	1.00 ± 0.00	Sown in lines with line spacing of 1 m
8	1.29 ± 0.02	0.83 ± 0.01	1.63 ± 0.07	1.00 ± 0.00	0.47 ± 0.04	1.00 ± 0.00	Sown in lines with line spacing of 1 m
10	1.32 ± 0.04	0.85 ± 0.02	1.52 ± 0.10	1.13 ± 0.13	0.50 ± 0.05	0.58 ± 0.03	1 × 1
16	1.61 ± 0.03	1.05 ± 0.02	1.43 ± 0.10	1.00 ± 0.00	0.58 ± 0.02	0.72 ± 0.03	1 × 2
30	1.87 ± 0.04	1.70 ± 0.02	1.87 ± 0.09	1.43 ± 0.07	0.77 ± 0.04	0.77 ± 0.03	1 × 2

**C**_NE-SW_, crown diameter in the northeast-southwest direction; C_NW-SE,_ crown diameter in the northwest-southeast direction; G_NE-SW_, ground diameter in the northeast-southwest direction; G_NW-SE_, ground diameter in the northwest-southeast direction. The values are the means ± SE (n = 3).

**Figure 4:**
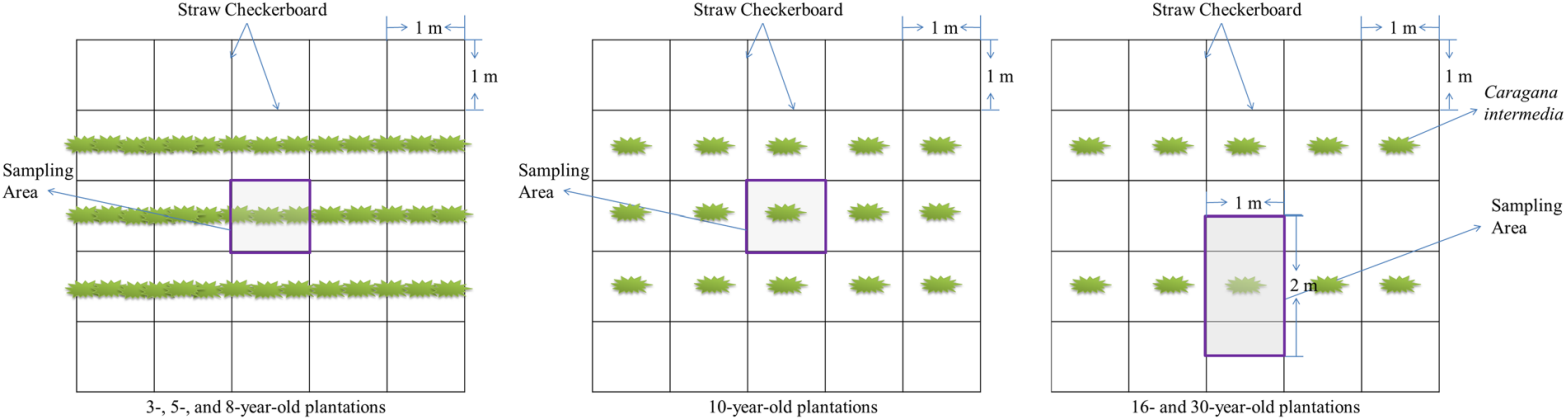
The sampling area of different ages for the *C. intermedia* plantations.

For the 3-, 5- and 8-year-old *C. intermedia* plantations, *C. intermedia* was sown in lines; therefore, we regarded a *C. intermedia* line in one straw checkerboard (1 × 1 m) as one standard shrub when we conducted experiments. Each harvested shrub was separated into the leaves, branches, stem bark, stem wood, and roots with diameters > 5 mm (roots > 5 mm), roots with diameters between 2 mm and 5 mm (2 mm < roots** ≤ **5 mm) and roots with diameters ≤ 2 mm (roots** ≤ **2 mm). Root samples were obtained by the total excavation of the standard shrubs extending out from the shrub centre to the middle of the lines and plants (Fig. 4). Therefore, the sampling area for the 3-, 5-, 8- and 10-year-old plantations was 1 m^2^, and the area for the 16- and 30-year-old plantations was 2 m^2^. The soil samples were sieved through a 0.6-mm sieve to obtain the roots. The sampling depth was 80 cm, at which point no more roots were visible. The fresh weights of all the components were measured *in situ*, and samples of every component from each standard shrub were oven-dried at 65 °C for 48 h and weighed to determine the ratio of fresh weight to dry biomass.

### Carbon concentration analysis

Dried samples of the different shrub components in each plantation were ground and sieved through a 100-mesh sieve, and the carbon concentrations were quantified using the induction furnace method with a CHNOS elemental analyser.

### Biomass carbon density and carbon accumulation rate calculation

The following equations were used to calculate the shrub biomass carbon density:

1 $$B=\sum _{{\rm{i}}}^{{\rm{m}}}\,{B}_{{\rm{it}}}/100\,{\rm{A}}$$

2 $$C=\sum _{{\rm{i}}}^{{\rm{m}}}\,{B}_{{\rm{i}}}\times {C}_{{\rm{i}}}$$

where *B* is the shrub biomass density (t·ha^−1^), *B*_it_ is the i component biomass of the whole standard shrub (g), *A* is the sampling area (m^2^), *C* is the biomass carbon density (t·ha^−1^), *B*_i_ is the i component biomass density (t·ha^−1^), and *C*_i_ is the i component biomass carbon concentration (%).

The following equation was used to calculate the shrub carbon accumulation rate:

3 $${C}_{r}=C/Y$$

where *C*_r_ is the shrub carbon accumulation rate (t·ha^−1^·yr^−1^) and *Y* is the stand age (yr).

### Data analysis

All the results are reported as the means (three measurements) ± standard error (SE). A one-way ANOVA was used to compare the biomass, carbon concentration, biomass carbon density and annual carbon sequestration among stands of different ages, and Duncan’s multiple range test was used to evaluate the differences among the means. The SPSS ver. 16.0 software was used for all the statistical analyses.

## Data Availability

The datasets analysed during the current study are available from the corresponding author upon reasonable request.

## References

[1] Dixon RK (1994). Carbon pools and flux of global forest ecosystems. Science.

[2] Keleş S (2010). Forest optimisation models including timber production and carbon sequestration values of forest ecosystems: a case study. Int. J. Sust. Dev. World.

[3] Wu J (2012). Effects of climate variability and functional changes on the interannual variation of the carbon balance in a temperate deciduous forest. Biogeosciences.

[4] Härkönen S, Lehtonen A, Eerikäinen K, Peltoniemi M, Mäkelä A (2011). Estimating forest carbon fluxes for large regions based on process-based modelling, NFI data and Landsat satellite images. For. Ecol. Manage..

[5] Sun XY, Wang GX, Huang M, Chang RY, Ran F (2016). Forest biomass carbon stocks and variation in Tibet’s carbon-dense forests from 2001 to 2050. Sci. Rep..

[6] Redondo-Brenes A (2007). Growth, carbon sequestration, and management of native tree plantations in humid regions of Costa Rica. New Forests.

[7] Losi CJ, Siccama TG, Condit R, Morales JE (2003). Analysis of alternative methods for estimating carbon stock in young tropical plantations. For. Ecol. Manage..

[8] Sean TC, Martin AR (2012). Carbon content of tree tissues:A synthesis. Forests.

[9] Li X (2011). Biomass and carbon storage in an age-sequence of Korea pine (*Pinus koraiensis*) plantation forests in central Korea. J. Plant Biol..

[10] Francis Justine, M., Yang, W. Q., Wu, F. Z. & Naeem Khan, M. Dynamics of Biomass and carbon sequestration across a chronosequence of masson pine plantations. *J. Geophys. Res-Biogeo*., 10.1002/2016JG003619 (2017).

[11] Fonseca W, Alice FE, Rey-Benayas JM (2012). Carbon accumulation in aboveground and belowground biomass and soil of different age native forest plantations in the humid tropical lowlands of Costa Rica. New Forests.

[12] Pooter H (2012). Biomass allocation toleaves, stems and roots: Meta-analyses of interspecific variation and environmental control. New Phytol..

[13] Hu HF, Wang ZH, Liu GH, Fu BJ (2006). Vegetation carbon storage of major shrublands in China. Journal of plant ecology.

[14] Gong CM (2016). Carbon Storage Patterns of Caragana korshinskii in Areas of Reduced Environmental Moisture on the Loess Plateau, China. Sci. Rep..

[15] Conti G, Enrico L, Casanoves F, Díaz S (2013). Shrub biomass estimation in the semiarid Chaco forest:a contribution to the quantification of an underrated carbon stock. Ann. For. Sci..

[16] Nosetto MD, Jobbágy EG, Paruelo JM (2006). Carbon sequestration in semi-arid rangelands:comparison of *Pinus ponderosa* plantations and grazing exclusion in NW Patagonia. J. Arid. Environ..

[17] Yu Y, Jia ZQ (2014). Changes in soil organic carbon and nitrogen capacities of *Salix cheilophila* Schneid along a revegetation chronosequence in semi-arid degraded sandy land of the Gonghe Basin, Tibet Plateau. Solid Earth.

[18] Perez-Quezada JF, Delpiano CA, Snyder KA, Johnson DA, Franck N (2011). Carbon pools in an arid shrubland in Chile under natural and afforested conditions. J. Arid. Environ..

[19] Zandler H, Brenning A, Samimi C (2015). Quantifying dwarf shrub biomass in an arid environment: comparing empirical methods in a high dimensional setting. Remote Sens. Enviro..

[20] Wang XP, Li XR, Xiao HL, Pan YX (2006). Evolutionary characteristics of the artificially revegetated shrub ecosystem in Tengger Desert, northern China. Ecol. Res..

[21] Houghton RA, Hall F, Goetz SJ (2009). Importance of biomass in the global carbon cycle. J Geophys. Res..

[22] Tao Y, Zhang YM (2013). Evaluation of vegetation biomass carbon storage in deserts of Central Asia. Arid Land Geography.

[23] Wang, J., Yao, Y. F., Guo, Y. F., Tang, J. & Chang, W. D. The Carbon Sink Dynamic Research on the *Caragana microphylla* and *Salix gordejevii* Shrub Ecosystem in the Southern Edge of Horqin Sandland. *Ecology and Environmental Sciences* **24**(11), 1785–1790 (2015).

[24] Su YZ, Zhang TH, Li YL, Wang F (2005). Changes in soil properties after establishment of *Artemisia halodendron* and *Caragana microphylla* on shifting sand dunes in semiarid Horqin Sandy Land, northern China. Environ. Manage..

[25] Jiang DM, Cao CY, Zhang Y, Cui ZB, Han XS (2014). Plantations of native shrub species restore soil microbial diversity in the Horqin Sandy Land, northeastern China. J. Arid Land.

[26] Li QX, Jia ZQ, Liu T, Feng LL, He LXZ (2017). Effects of different plantation types on soil properties after vegetation restoration in an alpine sandy land on the Tibetan Plateau, China. J. Arid Land.

[27] Vagen TG, Lal R, Singh BR (2005). Soil carbon sequestration in sub-Saharan Africa:a review. Land Degrad. Dev..

[28] Henderson DC, Chapman R (2006). *Caragana arborescens* Invasion in Elk Island National Park, Canada. Nat. Area J..

[29] Zhang TH, Su YZ, Cui JY, Zhang ZH, Chang XX (2006). A Leguminous Shrub (*Caragana microphylla*) in Semiarid Sandy Soils of North China. Pedosphere.

[30] Bhardwaj PK, Ahuja PS, Kumar S (2010). Characterization of gene expression of QM from *Caragana jubata*, a plant species that grows under extreme cold. Mol. Biol. Rep..

[31] Zhu J (2013). Reference Gene Selection for Quantitative Real-time PCR Normalization in *Caragana intermedia* under Different Abiotic Stress Conditions. Plos One.

[32] Zhao YZ (2005). The distribution pattern and ecological adaptation of *Caragana microphylla*. C.davazamcii and C. korshinskii. Acta ecologica sinica.

[33] Zhang, D. S. *et al*. *Sandy Desertification and it’s Control in the Qinghai Plateau*. Beijing: Science Press 6–40 (2009).

[34] Lu Q, Wang XQ, Wu B, Yang HX (2009). Can mobile sandy land be vegetated in the cold and dry Tibetan Plateau in China?. Frontiers of Biology in China.

[35] Li QX (2014). Effects of soil improvement of *Caragana intermedia* plantations in alpine sandy land on Tibet Plateau. Acta Ecologica Sinica.

[36] Li QX (2015). Spatial heterogeneity of soil nutrients after the establishment of *Caragana intermedia* plantation on sand dunes in alpine sandy land of the Tibet Plateau. Plos one.

[37] Liu LY (2012). Water use strategy of different stand ages of *Caragana intermedia* in alpine sand land. Journal of Arid Land Resources and Environment.

[38] Jia ZQ, Zhu YJ, Liu LY (2012). Different water use strategies of juvenile and adult *Caragana intermedia* plantations in the Gonghe Basin, Tibet Plateau. Plos One.

[39] Liu LY (2012). Root system distribution of *C.intermedia* plantation in Gonghe Basin, Qinghai Province. Journal of desert research.

[40] Pilli R, Anfodillo T, Carrer M (2006). Towards a functional and simplified allometry for estimating forest biomass. For. Ecol. Manage..

[41] Cao J, Wang X, Tian Y, Wen Z, Zha T (2012). Pattern of carbon allocation across three different stages of stand development of a Chinese pine (*Pinus tabulaeformis*) forest. Ecol. Res..

[42] Deng, L., Han, Q., Zhang, C., Tang, Z. S. & Shangguan, Z. P. Above-Ground and Below-Ground Ecosystem Biomass Accumulation and Carbon Sequestration with *Caragana korshinskii* Kom Plantation Development. *Land Degrad. Dev.*, 10.1002/ldr.2642 (2017).

[43] Wang X, Yang DG, Xiong HG, Liu YY, Gong YM (2016). Characteristics of biomass carbon and soil organic carbon of 8 common shrubs in arid regions of Xinjiang. Chinese. Journal of Ecology.

[44] Singh G, Singh B (2017). Biomass equations and assessment of carbon stock of *Calligonum polygonoides* L. a shrub of Indian arid zone. Curr. Sci. India.

[45] Osone Y, Tateno M (2005). Applicability and limitations of optimal biomass allocation models:a test of two species from fertile and infertile habitats. Ann. Bot-london.

[46] Qiu Y (2015). Biomass estimation modeling and adaptability analysis of organ allocation in six common shrub species in Tianshan Mountains forests, China. Acta Ecologica Sinica.

[47] Bert D, Danjon F (2006). Carbon concentration variations in the roots, stem and crown of mature *Pinus pinaster* (Ait.). For. Ecol. Manage..

[48] Maiti, R., Rodriguez, H. G. & Kumari, C. A. Trees and Shrubs with High Carbon Fixation/Concentration. *Forest Research*, 10.4172/2168-9776.S1-003 (2015).

